# Peer Victimization Influences Attention Processing Beyond the Effects of Childhood Maltreatment by Caregivers

**DOI:** 10.3389/fpsyg.2022.784147

**Published:** 2022-03-04

**Authors:** Benjamin Iffland, Frank Neuner

**Affiliations:** Department of Psychology, Bielefeld University, Bielefeld, Germany

**Keywords:** child maltreatment, peer victimization, attentional bias, emotional Stroop, dot-probe

## Abstract

**Background:**

Different types of maltreatment (emotional, physical, and sexual) lead to distortions in emotion and attention processing. The present study investigated whether the experience of peer victimization in childhood and adolescence has an additional influence on attention processing in adulthood.

**Methods:**

Two non-clinical samples consisting of individuals with different levels of experiences of maltreatment were recruited. In an evaluative conditioning task, images of faces with neutral emotional expression were either associated with short videos of intense negative statements, or associated with neutral videos. Subsequently, these faces were used as stimuli in an emotional Stroop task as well as a dot-probe task.

**Results:**

In both tasks, hierarchical regression analyses revealed that retrospective reports of relational peer victimization made an incremental contribution to the prediction of attentional biases beyond child maltreatment. In the emotional Stroop task, emotional abuse was the strongest predictor for an attentional bias showing delayed responses to negatively associated faces, while peer victimization was associated with faster responses to negatively associated faces. In the dot-probe task, relational peer victimization was the strongest predictor for an attentional bias. When the attentional bias was examined in more detail, though, peer victimization did not show incremental contributions although emotional abuse remained the strongest predictor for facilitated attention toward negatively associated neutral faces.

**Conclusion:**

Experiences of peer victimization leave additional cognitive scars beyond effects of childhood maltreatment by caregivers. It is likely that attentional biases in the aftermath of victimization put individuals at risk for the development of psychopathology.

## Introduction

Attentional biases are characterized by a selective and differential allocation of attention toward emotional stimuli in comparison to neutral stimuli (for a review, see Cisler and Koster, 2010). Specifically, attentional biases can be divided into facilitated attention (i.e., faster detection of threatening stimuli), difficulty in disengagement (i.e., disengaging attention from threat stimuli is more difficult than disengaging from neutral stimuli), and attentional avoidance (i.e., shifting attention toward locations opposite the location of threat; Cisler and Koster, 2010). In addition to a large body of research showing that attentional biases robustly emerge among clinical populations (e.g., Bar-Haim et al., 2007; Cisler and Koster, 2010), studies indicated that experiences of childhood maltreatment are also associated with altered attentional processes in the processing of threatening information (e.g., Pollak et al., 2001; Grant et al., 2011; Günther et al., 2015). However, the kinds of attentional biases that have been linked to childhood maltreatment have differed across studies. For example, individuals reporting a history of childhood maltreatment showed an enhanced sensitivity to detect threatening cues from emotionally ambiguous faces which was thought to be indicative of a facilitated processing of threatening information (Pollak and Sinha, 2002; Gibb et al., 2009). Attentional avoidance of threatening faces and difficulties in disengaging from sad faces, though, have been reported in samples of children who had experienced physically abuse (Pine et al., 2005; Romens and Pollak, 2012). Since childhood maltreatment is a heterogenous phenomenon that includes various types of abuse and neglect, it is likely to assume that different forms of maltreatment, such as abuse and neglect, influence attentional biases toward threatening stimuli differently. Similarly, various kinds of childhood maltreatment have differential psychopathological outcomes (Danielson et al., 2005; Teicher et al., 2006; Lobbestael et al., 2010; Teicher and Samson, 2013). This is also supported by recent reports of differential effects of abuse and neglect on neural mechanisms that may link childhood maltreatment to psychopathology and alterations in emotional functioning (Dong et al., 2004; McLaughlin et al., 2014; Sheridan and McLaughlin, 2014; Humphreys and Zeanah, 2015; Zeanah and Sonuga-Barke, 2016; Roth et al., 2018). Critically, however, there are a limited number of studies examining differential effects of specific kinds of childhood maltreatment on attentional biases.

In one study of childhood maltreatment and attentional biases, Günther et al. (2015) analyzed the differential impact of five factors of childhood maltreatment (emotional abuse, emotional neglect, physical abuse, physical neglect, and sexual abuse) on attentional biases in a dot-probe task and reported that sustained attention toward sad facial expressions was associated with emotional forms of maltreatment and physical neglect, but not with physical and sexual abuse. Notably, the relationship of childhood maltreatment and sustained attention was not confounded by the severity of symptoms of depression, even though a sample of depressed individuals was examined. Similarly, attentional processes varied as a function of different forms of childhood maltreatment in a study using a visual search paradigm combining a social conditioning paradigm with a face in the crowd recognition task (Iffland and Neuner, 2020). Specifically, emotional forms of maltreatment were particularly associated with an altered sensitivity in detecting faces. While emotional abuse was associated with faster recognition of negatively associated faces, emotional neglect was related to slower detection of both negative and neutral faces. Experiences of physical abuse were shown to be associated with slower detection of negatively associated faces compared to neutrally associated faces. Past experiences of sexual abuse, however, did not have an impact on individuals’ performance in this study (Iffland and Neuner, 2020). In addition, processing of emotional cues varied between types of maltreatment in a facial emotion recognition task (Pollak et al., 2000). In this study, physically abused children showed a response bias for angry facial expressions, whereas physically neglected children presented with difficulties in differentiating among emotional expressions. Reports of differential effects of maltreatment types on attentional processes were not supported by Fani et al. (2011), though. In their study with a sample of patients with posttraumatic stress disorder (PTSD), childhood maltreatment uniquely predicted attentional biases toward happy faces relative to neutral faces. However, differing associations between attention processing and different types of childhood maltreatment were not found. Although there is growing body of literature indicating that different types of maltreatment affect attentional biases differently, further research is needed to examine the extent to which the various kinds of maltreatment account for differences in attentional processes.

In addition, most studies examined attentional biases as a function of childhood maltreatment including emotional, physical, or sexual abuse and neglect by caretakers (e.g., Günther et al., 2015; Iffland and Neuner, 2020). However, maltreatment is not isolated within the context of families alone. There are also experiences of maltreatment that involve emotional forms of abuse by peers (Storch et al., 2005; Siegel et al., 2009). This relational peer victimization is characterized by bullying, verbal threats or aggression, malicious manipulation of a relationship, friendship withdrawal, and damaging another’s peer relationships (Siegel et al., 2009). Prevalence rates of repeated peer victimizations range between 10 to 20% in school children, with periodic adversities being indicated even more frequently (e.g., Rudolph et al., 2010). Similar to experiences of maltreatment by caretakers, a history of relational peer victimization increases the risk of various forms of psychopathology, with peer victimization predicting psychological symptoms even beyond the effects of child maltreatment (Storch et al., 2005; Sansen et al., 2014). For instance, it has been demonstrated that emotional peer abuse is associated with increased rates of depression, anxiety disorders, suicidality, psychosomatic complaints, sleep and eating disorders, self-injurious behavior, dissociation, substance use, and psychosis (e.g., Gladstone et al., 2006; Teicher et al., 2010; Copeland et al., 2015). In particular, the relationship of peer victimization to social anxiety is well established (e.g., Storch et al., 2003; Storch and Masia-Warner, 2004; Ranta et al., 2009; Siegel et al., 2009; Iffland et al., 2012; Sansen et al., 2015).

With respect to attention processes, children who reported more frequent experiences of peer victimization showed less interference when confronted with victim-related words in an emotional Stroop task (Rosen et al., 2007). Accordingly, within the framework of a modified social-information-processing model, the authors proposed that peer victimization was associated with preemptive, defensive processing of threatening cues (Rosen et al., 2007). On the contrary, individuals who had experienced peer victimization showed delayed responses in color-naming negative adjectives compared to neutral adjectives in an emotional Stroop task (Iffland et al., 2019). However, in line with Rosen et al. (2007), an additional dot-probe task applied in the same sample revealed that participants with a history of peer victimization avoided negative adjectives rather than detecting them faster or allocating their attention toward negative words. Even more, since the pattern of results did not differ between negative and positive adjectives, the results of this study indicated that peer victimized individuals presented with a general emotion-avoidant, rather than threat avoidant, attentional style (Iffland et al., 2019). Most notably, in this sample of psychiatric inpatients and healthy controls, attentional avoidance of emotional words was more closely associated with experiences of peer victimization than with the current diagnostic status (Iffland et al., 2019). It may be that attentional avoidance of emotional stimuli could increase the risk of victims of peer abuse for the development of psychopathology.

Recent studies in clinical as well as healthy samples suggest that negative life experiences influence the magnitude of attentional biases (Field et al., 2001; Gibb et al., 2009; Aishu and Chunmei, 2014; Günther et al., 2015; Iffland et al., 2019). Indeed, attentional biases may play a crucial role in the link between childhood maltreatment experiences and the development of psychopathology (Pollak, 2003). Still, knowledge about the unique contributions of different forms of childhood maltreatment to the development of attentional biases is scarce. Moreover, to our knowledge, there are no studies examining the incremental effect of relational peer victimization on attentional biases when controlling for experiences of child maltreatment. Therefore, the purpose of the current sample was to extend the previous research (e.g., Günther et al., 2015; Iffland and Neuner, 2020) by examining differential associations between various types of childhood maltreatment and peer victimization and attentional biases in healthy adult samples.

As experimental tasks to measure attentional biases, we applied both an emotional Stroop task (Stroop, 1935) and a dot-probe task (MacLeod et al., 1986; Koster et al., 2004; for a review see Cisler and Koster, 2010). Following previous studies (Rosen et al., 2007; Iffland et al., 2019), we decided to use the emotional Stroop task. The emotional Stroop task is the most commonly used task to measure attentional biases indicating interference by higher response times to color-naming of threat words compared to neutral words (Cisler and Koster, 2010). Because of several shortcomings in the interpretation of attentional biases measured with the emotional Stroop task (Cisler and Koster, 2010), however, the dot-probe task was additionally used. The advantage of the dot-probe task is that is was developed to distinguish different aspects of attentional biases, i.e., difficulty in disengaging and facilitated attention (Cisler and Koster, 2010). Moreover, the dot-probe task allows for the measurement of spatial attention allocation (MacLeod et al., 1986). In line with a previous study extending the evolutionary theory of attentional biases (Iffland and Neuner, 2020), we postulated that not only the detection of emotional facial expressions, but also the detection of differently evaluated individuals, represented by neutral faces that are associated with different emotions, may be shaped by life experiences differently. That is, threat is not always linked to overt facial expressions of negative emotions in real-world settings. Thus, rather than perceptual features of their faces, the rapid identification of potential perpetrators should initiate a quick location, recognition, and response to potential social threats (Iffland and Neuner, 2020). And the rapid identification should depend on previous experiences with specific persons (e.g., childhood maltreatment). Therefore, we applied a more ecologically valid test for attentional biases in maltreated individuals by combining an evaluative conditioning task with an emotional Stroop and a dot-probe task. As a first step, we coupled still images of neutral faces with short videos of negative/disapproving evaluations vs. neutral statements of the same actors from the E.Vids video set in the evaluative conditioning task (Blechert et al., 2013; see also Iffland and Neuner, 2020). Second, the images of neutral faces were used as stimuli in the emotional Stroop and dot-probe tasks. By doing this, we aimed at extending previous research using a visual search paradigm (Iffland and Neuner, 2020). Response times (RTs) were used to detect whether neutral faces evaluated as being negative were associated with different attention processes than neutral faces with a neutral evaluation.

The aim of the present study was to examine differential unique contributions of various forms of childhood maltreatment to attentional biases in facial emotion processing. Particularly, extending previous research (e.g., Günther et al., 2015; Iffland and Neuner, 2020), the incremental contribution of experiences of peer victimization in the prediction of attentional biases when controlling for histories of childhood maltreatment was assessed. With respect to previous studies reporting that peer victimization predicts psychopathology beyond the effects of childhood maltreatment (Sansen et al., 2014), we hypothesized that peer victimization would make a significant incremental contribution of variance to the prediction of attentional biases. Specifically, in line with a previous study (Iffland et al., 2019), we assumed that peer victimization would be associated with attentional avoidance of negatively associated faces. Regarding the unique contributions of forms of childhood maltreatment by caretakers, in line with previous studies (Günther et al., 2015; Iffland and Neuner, 2020), we assumed that attentional biases would be particularly associated with emotional forms of childhood maltreatment.

## Materials and Methods

### Participants

Two samples were recruited to address the study’s aims. The first sample completed the emotional Stroop task and the second completed the dot-probe task. Participants of both samples were recruited through online advertisements and bulletins on the Bielefeld University campus advertising a study examining the consequences of personality traits on attention. Inclusion criteria were age between the ages of 18 and 65 and sufficient knowledge of German language (clearly able to understand the information and instructions). No further exclusion criteria were applied. The emotional Stroop task sample consisted of 94 participants (54 females, 57.4%) ranging in age from 18 to 65 years with a mean of 26.40 (*SD* = 10.65). The dot-probe task sample consisted of 89 participants (56 females, 62.9%) ranging in age from 18 to 60 years with a mean of 25.10 (*SD* = 7.44). In both samples, each participant read and signed an informed consent form that was approved by the Ethics Committee of Bielefeld University. The demographic characteristics of the samples and participants’ means on the assessments are presented in Table 1.

**TABLE 1:** Subject characteristics and mean values on the assessments.

	Emotional Stroop task (*N* = 94)	Dot-probe task (*N* = 89)
Age, *M* (*SD, range*)	26.40 (10.65, 18–65)	25.10 (7.44, 18–60)
Gender,% *female* (*n*)	57.4 (54)	62.9 (56)
Family status,% *single* (*n*)	57.4 (54)	49.4 (44)
Educational level,% high school graduation and higher (*n*)	83.0 (78)	88.7 (79)
Symptoms of Depression^1^, *M* (*SD*)	8.83 (6.23)	8.97 (5.22)
General Psychopathology^2^, *M* (*SD*)	0.48 (0.38)	0.50 (0.40)
Trait Anxiety^3^, *M* (*SD*)	39.30 (11.60)	41.05 (9.54)
Childhood Trauma Questionnaire, *M* (*SD*)	34.59 (9.76)	35.16 (11.35)
Emotional Abuse, *M* (*SD*)	7.87 (3.24)	8.21 (4.14)
Emotional Neglect, *M* (*SD*)	9.05 (3.60)	8.96 (3.99)
Physical Abuse, *M* (*SD*)	5.64 (2.30)	5.79 (2.04)
Physical Neglect, *M* (*SD*)	6.61 (2.06)	7.04 (2.65)
Sexual Abuse, *M* (*SD*)	5.41 (1.28)	5.16 (0.82)
Peer Victimization^4^, *M* (*SD*)	9.52 (6.96)	9.13 (5.71)

*^1^Beck Depression Inventory; ^2^Brief Symptom Inventory — Global Severity Index; ^3^State Trait Anxiety Inventory-Trait; ^4^Fragebogen zu belastenden Sozialerfahrungen.*

### Stimuli, Design, and Apparatus

In line with a previous study (Iffland and Neuner, 2020), we utilized a social conditioning paradigm using 3000 ms duration videos of negative and neutral sentences from the E.Vids video set (Blechert et al., 2013) for the conditioning of neutral faces to negative/disapproving vs. neutral valence. Within this paradigm, still images of neutral faces from four different actors (two female) served as conditioned stimuli (CSs) predicting dynamic videos of negative/disapproving evaluations (e.g., ‘You’re ridiculous,’ ‘I hate you,’ ‘I can’t stand you’) vs. neutral statements (e.g., ‘The bus is stopping,’ ‘It’s windy outside,’ ‘It’s 4 o’clock’) of the same actors as unconditioned stimuli (US) (for details see Wiggert et al., 2017). No information about CS-US contingencies was provided. The conditioning consisted of 64 trials, 32 trials (16 per actor) coupling CSs with a negative US and 32 trials (16 per actor) coupling CSs with a neutral US. Each of the four actors spoke eight different sentences, each sentence presented twice. For each participant, two actors were presented in the socially negative and two actors were presented in the neutral condition. Actors’ conditions were counterbalanced over participants. Video volume was constant across participants. Each trial started with the presentation of a black fixation cross in the center of a white screen for 500 ms before being replaced by the CS. Conditioned stimuli were shown for 1000 ms and were followed by the presentation of a black fixation cross in the center of a white screen for 1500 ms. Then, the USs were presented for 3000 ms. Inter-trial intervals varied randomly between 5000 and 7000 ms. Stimuli were presented on a 23-inch LCD monitor with a resolution of 1920 × 1080 pixels and 120 Hz refresh rate, using E-Prime 2.0 (Psychology Software Tools, Inc., Sharpsburg, PA, United States).

For both the emotional Stroop and the dot-probe task, we used the four still images of neutral faces that were negatively or neutrally associated in the social conditioning paradigm.

Participants viewed stimuli at a distance of 60 cm. Stimuli were presented on a 23-inch LCD monitor with a resolution of 1920 × 1080 pixels and 120 Hz refresh rate. We used the software package Inquisit 4.0.3 (Millisecond Software, Seattle, WA, United States) to deliver stimuli and record responses and reaction times (RTs).

The emotional Stroop task consisted of 128 trials. In total, 64 negatively and 64 neutrally associated neutral faces were shown, in each case 16 faces were colored in red, 16 in blue, 16 in green, and 16 were colored in yellow. For this purpose, we used black-and-white (binary) images of the faces, in which the white parts were colored in the respective colors. Each single face was presented 32 times, eight times in each color. Each image was 20.5 cm (width) × 18 cm (height) and presented on a black background. Stimuli were shown throughout until the participants responded. After an intertrial interval of 200 ms the next stimulus was presented. Responses were made on an external keyboard in which four keys were activated. The participants’ task was to identify the color of the presented faces as quickly and as accurately as possible. Participants indicated their response by pressing buttons on an external keyboard with the index and middle fingers of both hands. In order to ensure that the participants were able to assign the colors to the appropriate buttons, the assignment of buttons and colors was presented on the screen throughout the experiment. The assignment of buttons was counterbalanced across participants. The order of faces, face valences, and colors was randomized. No feedback on accuracy was provided.

The dot-probe task consisted of two blocks of 64 trials each, with a short break between the blocks. There were two different types of trials in the present task: negative–neutral and neutral-neutral, with negatively and neutrally, and neutrally and neutrally associated neutral faces combined, respectively. For each trial, two faces were presented simultaneously. The face pairs were presented with one face beside the other (horizontal) in the middle of the screen. Each image was 8 cm (width) × 6.7 cm (height). The dot-probe experiment began with 12 practice trials using neutral–neutral face pairs to familiarize participants with the task. Each trial started with a black fixation cross presented in the center of a white screen for 500 ms. Then, a face pair appeared with one face beside the other for 500 ms. A gray dot emerged in one of the face locations immediately after the offset of the faces. The location of the target face (left or right) and probe (left or right) was randomized for all trials. The inter-trial interval for all trials was 500 ms. Responses were made on an external keyboard in which two keys were activated. Participants were instructed to respond as quickly and as accurately as possible and to indicate the location of the gray dot (left or right) by pressing either the “E” (left) or “I” (right) keys on an external keyboard with the index fingers of both hands. The two types of face pairs were randomly formed. Each face was presented 64 times (32 times on each side) for a total of 128 experimental trials. The combination and order of face pairs varied randomly for each participant. No feedback on accuracy was provided.

### Instruments

The *Fragebogen zu belastenden Sozialerfahrungen* (*FBS;* [Adverse Social Experiences Questionnaire]) was used to assess relational peer victimization (Sansen et al., 2013). This self-report questionnaire consists of 22 items describing aversive social situations like rejection, exclusion, being laughed at, insulted, and teased by peers (e.g., “I was excluded from games or activities by other children or adolescents,” “I have been laughed at in the presence of other children”). For each situation, respondents were asked whether or not they have experienced this situation during childhood (age 6–12) or adolescence (age 13–18). The total score was calculated as a sum of “Yes” responses across both age periods and ranged from 0 to 44. The total-score of the FBS presented with a satisfying stability over a 20-month period (*r* = 0.89) (Sansen et al., 2013). Moderate correlations with the scales of the Childhood Trauma Questionnaire (Wingenfeld et al., 2010), as well as an incremental contribution to the prediction of psychopathology, support the idea that the FBS assesses an additional construct of child maltreatment (Iffland et al., 2012; Sansen et al., 2013).

Childhood maltreatment was measured using the German Version of the Childhood Trauma Questionnaire (CTQ; Wingenfeld et al., 2010; Klinitzke et al., 2012). With the CTQ, all common types of childhood maltreatment (emotional abuse, emotional neglect, physical abuse, physical neglect, and sexual abuse) that have occurred before the age of 18 can be assessed. In the present study, dimensional sum scores for each CTQ subscale were used in the statistical analyses. The CTQ physical neglect subscale was not included in the following statistical analyses because it was highly correlated with the other CTQ subscales, and presented with a weak internal consistency in comparison to the other subscales in a validation study (Klinitzke et al., 2012). For the sake of a comparison with other samples, however, mean score and frequency of the CTQ physical neglect subscale are presented. Table 2 presents the bivariate Pearson correlation coefficients of different types of maltreatment and peer victimization for both samples.

**TABLE 2:** Bivariate Pearson correlation coefficients of different types of maltreatment and peer victimization for samples of the emotional Stroop (*N* = 94) and the dot-probe task (*N* = 89).

Emotional Stroop task	Emotional abuse	Emotional neglect	Physical abuse	Sexual abuse	Peer victimization
	*r*	*R*	*r*	*r*	*r*
Emotional abuse	–				
Emotional neglect	0.68***	–			
Physical abuse	0.47***	0.45***	–		
Sexual abuse	0.56***	0.49***	0.50***	–	
Peer victimization	0.37***	0.51***	0.42***	0.23*	–

**Dot-probe task**	**Emotional abuse**	**Emotional neglect**	**Physical abuse**	**Sexual abuse**	**Peer victimization**
	***r***	***R***	***r***	***r***	***R***

Emotional abuse	–				
Emotional neglect	0.74***	–			
Physical abuse	0.71***	0.53***	–		
Sexual abuse	0.43***	0.45***	0.27**	–	
Peer victimization	0.49***	0.45***	0.28**	0.38***	–

*p < 0.05, **p < 0.01, ***p < 0.001.

Moreover, the assessment battery included a socio-demographic questionnaire as well as well-established questionnaires for symptoms of depression (German version of the Beck Depression Inventory II, BDI-II; Hautzinger et al., 2006; Kühner et al., 2007), general psychopathology and psychological distress (German version of the Brief Symptom Inventory, BSI; Derogatis and Melisaratos, 1983; Derogatis, 1993; Franke, 2000), and trait anxiety (German version of the State Trait Anxiety Inventory-Trait, STAI-T; Spielberger et al., 1970; Laux et al., 1981).

### Procedure

Procedures were identical in both tasks. Prior to the laboratory session, participants were asked to complete the assessment battery described above. Afterward, participants were tested individually in a darkened room. All instructions for the tasks were presented on the computer screen for the participants to read. Participants were informed that they would see a series of images and videos of different people and they would be asked to evaluate them. During a pre-conditioning rating phase, subjects evaluated neutral still images of the actors (for details see Wiggert et al., 2017) for valence, arousal, and disapproval using an on-screen visual analog scale to control for baseline differences in the evaluation of the four actors that were presented in the social conditioning paradigm. Next, participants attended to the 64 trials of the social conditioning paradigm followed by a post-conditioning evaluative rating phase of each actor’s still image using the same rating scales described above. Post-conditioning evaluative rating served to evaluate whether social conditioning was successful. Next, participants completed either the emotional Stroop tasks or the dot-probe task. Before the emotional Stroop task, participants were informed that they would see faces presented in different colors and that their task was to identify the color of the presented faces. Participants indicated their response by pressing either the button “D,” “F,” “J,” or “K” on a keyboard with their index and middle fingers of both hands. Assignment of buttons to colors alternated between participants. Before the dot-probe task, participants were informed that they would see pairs of faces and that their task was to indicate the location of the gray dot (left or right). Participants indicated their response by pressing either the button “E” (left) or “I” (right) on a keyboard with the index fingers of both hands. Following completion of the attention tasks, another rating phase was completed using the same steps as the pre- and post-conditioning phase. This was used to examine if the effect of social conditioning lasted throughout the attention tasks. After these tasks were completed, participants were debriefed.

### Data Reduction

In the emotional Stroop task an attentional bias was indicated by greater color-naming latencies following negatively associated faces in comparison with neutrally associated faces (Williams et al., 1996). Therefore, a difference score for the reaction times (RT) in color-naming negative and neutral trials was calculated (emotional Stroop Index = RT negatively associated faces – RT neutrally associated faces). Positive scores indicate a greater attentional bias in the processing of negatively associated faces. Furthermore, RTs of trials with negatively associated faces and trials of neutrally associated faces can be used to identify underlying mechanisms of attentional biases. That is, positive emotional Stroop index scores could be either caused by higher RTs in color-naming negatively associated faces or by lower RTs in color-naming neutrally associated faces. Initially, 95 participants were tested using the emotional Stroop task. Consistent with procedures of prior studies, trials with reaction times lower than 300 ms or higher than 4000 ms were excluded from analyses (Moritz et al., 2008; Wittekind et al., 2010). In addition, trials where participants indicated the wrong color (error trials) were excluded. Over 128 trials, participants indicated between 0 and 13 wrong colors (*M* = 5.96, *SD* = 4.81). No participants were excluded due to error rates higher than 25%. Outliers were defined as participants presenting mean reaction times that deviated more than three SDs from sample mean reaction times and were removed from analyses (*n* = 1). Accordingly, the remaining sample for the analyses of the emotional Stroop task consisted of 94 participants. Further, individual RT outliers, defined as ±2 SDs from the individual’s mean (4.7% of all correct trials), were excluded from the calculation of that participant’s mean score.

In the dot-probe task an attentional bias was indicated by either lower RTs to the probe if it emerged at the location where the participants were focusing their attention, or higher RTs to the probe when it appears in the location where the participants were not attending (Roberts et al., 2010). The attentional bias scores were calculated by subtracting participants’ RTs to the probe when it appeared in the same position as the target face (congruent trials) from participants’ RTs to the probe when it did not appear in the same position as the target face (incongruent trials; MacLeod and Mathews, 1988; Roberts et al., 2010). In the present study, the target faces were the negatively associated faces in the negative–neutral trials. According to previous research (MacLeod and Mathews, 1988; Roberts et al., 2010), significant positive bias scores indicate that participants were focusing their attention on the area around the target faces when the probe occurred, whereas significant negative bias scores indicate that participants were not attending to the area around the target faces when the probe occurred (i.e., avoidance).

To differentiate the mechanisms underlying the attentional bias (vigilance vs. difficulty to disengage), additional index scores were calculated (Koster et al., 2004). Vigilance should lead to faster responses on trials where the probe appeared where participants were attending compared to neutral trials. Difficulties in disengaging attention from negatively associated faces would result in slower reaction times on trials where the probe appeared in a location they were not attending to due to the time needed to shift attention from the negatively valenced location to the neutral location. Specifically, the Orienting Index score was calculated by subtracting participants’ RTs to the probe when it occurred in the same position as the target face (congruent trials) from participants’ RTs to the probe when two neutrally associated faces were presented (neutral trials; Koster et al., 2004). The Disengaging Index score was calculated by subtracting participants’ RTs to the probe when two neutrally associated faces were presented (neutral trials) from participants’ RTs to the probe when it did not occur in the same position as the target face (incongruent trials; Koster et al., 2004).

Initially, 94 participants were tested using the dot-probe task. In line with previous studies, trials with reaction times lower than 150 ms or higher than 2000 ms were excluded from analyses (Koster et al., 2004; Dewitte et al., 2007; Bardel et al., 2013). In addition, trials where participants indicated the incorrect location of the probe (error trials) were excluded. Out of 128 trials, participants indicated between 0 and 53 incorrect locations (*M* = 11.38, *SD* = 12.43). One participant was excluded due to error rates greater than 25%. Outliers were defined as participants presenting mean reaction times that deviated more than three SDs from mean reaction times and were removed from analyses (*n* = 4). Accordingly, the remaining sample for the analyses of the dot-probe task consisted of 89 participants. Moreover, individual RT outliers, defined as ±2 SDs from the individual’s mean (5.6% of all correct trials), were excluded from the calculation of that participant’s mean score.

### Statistical Analyses

For the planned multiple linear regression analyses using RTs as outcome variable, a statistical power analysis was performed for sample size estimation using G*Power 3.1 (Faul et al., 2009). With respect to previous results (Günther et al., 2015; Iffland and Neuner, 2020), the effect size (ES) in this study was considered to be medium to large using Cohen’s (1988) criteria (Cohen’s *f*^2^ = 0.25). With an α = 0.05, power = 0.95, and inclusion of seven predictors (emotional abuse, emotional neglect, physical abuse, sexual abuse, peer victimization, age, gender), the projected sample size needed with this ES was *N* = 86. We anticipated a loss of data of approximately 10 percent due to error trials and outliers. Therefore, we aimed at recruiting 94 participants for each attention task.

All statistical analyses were carried out using the Statistical Package for the Social Sciences (SPSS) 27. Consistent with procedures utilized in prior research (Iffland et al., 2018; Iffland and Neuner, 2020), we calculated experiential rating composite scores (mean-score of the arousal, valence, and disapproval ratings) for analyses of differential conditioning effects on self-report data in both samples. Experiential data was assessed through a 2 (CS-type: CS-negative, CS-neutral) x 3 (time of assessment: pre-conditioning, post-conditioning, post attention task) analysis of variance (ANOVAs) with repeated measures on CS-type and time of assessment. When necessary, additional *post hoc t*-tests were conducted separately for different times of assessment. When Mauchly’s test indicated violation of the sphericity assumption, Greenhouse–Geisser corrections were applied and the original degrees of freedom together with Greenhouse–Geisser ε are reported.

In order to determine the relative contribution of child maltreatment and relational peer victimization for the prediction of attentional bias indices, several sets of hierarchical multiple regression analysis were conducted. For this purpose, we used the continuous sum scores of the CTQ subscales emotional abuse, emotional neglect, physical abuse, and sexual abuse as well as the continuous sum score of the FBS. In the hierarchical regression analyses, age and gender were included as predictors in a first step. In a second step, emotional abuse, emotional neglect, physical abuse, and sexual abuse were added. Relational peer victimization was added in a third step. In the emotional Stroop task sample, regression analyses were conducted separately for the emotional Stroop index score and RTs of trials with negatively associated faces and trials of neutrally associated faces. Here, preliminary analyses showed no violation of the assumption of multicollinearity (tolerances > 0.40; variance inflation factors < 2.48). In the dot-probe task sample, hierarchical regression analyses were conducted separately for each of the three indices of the dot-probe task presented above. Preliminary analyses showed no violation of the assumption of multicollinearity in this sample (tolerances > 0.29; variance inflation factors < 3.50).

## Results

### Emotional Stroop

#### Social-Conditioning Paradigm

A CS-type × Time of assessment ANOVA using the experiential rating composite score was conducted to test whether the association of the neutral faces with negative and neutral valences was successful. The ANOVA revealed significant main effects of CS-type and time of assessment [CS-type: *F*(1,93) = 93.15; *p* < 0.001; η^2^ = 0.500; time of assessment: *F*(2,186) = 78.98; *p* < 0.001; η^2^ = 0.459; ε = 0.87]. Moreover, a significant interaction of CS-type and time of assessment was found [*F*(2,186) = 40.46; *p* < 0.001; η^2^ = 0.303; ε = 0.88]. While there were no significant differences in experiential ratings of the neutral faces before the conditioning task [*t*(93) = 1.36, *p* = 0.176], *post hoc t*-tests showed that experiential ratings of the negatively associated familiar faces were rated significantly more negative than the neutrally associated familiar faces immediately after the conditioning task as well as after the emotional Stroop task [post-conditioning: *t*(93) = 10.85, *p* < 0.001; post emotional Stroop task: *t*(93) = 5.44, *p* < 0.001]. Means and standard deviations of experiential rating scores as well as the experiential ratings of arousal, valence, and disapproval are presented in Table 3.

**TABLE 3:** Means and standard deviations on the experiential rating composite scores and the experiential ratings of arousal, valence, and disapproval for the emotional Stroop (*N* = 94) and the dot-probe task (*N* = 89).

	Pre-conditioning	Post-conditioning	Post attention task
**Emotional Stroop task**			
*Experiential rating composite score*			
Negatively associated neutral faces, *M* (*SD*)	42.97^a^ (10.29)	71.32^a^ (17.86)	61.23^a^ (18.49)
Neutrally associated neutral faces, *M* (*SD*)	44.81^a^ (10.14)	38.25^b^ (15.88)	41.06^b^ (20.23)
*Arousal*			
Negatively associated neutral faces, *M* (*SD*)	42.72^a^ (11.67)	66.31^a^ (20.94)	58.05^a^ (18.26)
Neutrally associated neutral faces, *M* (*SD*)	43.22^a^ (11.70)	34.04^b^ (17.38)	38.41^b^ (20.72)
*Valence*			
Negatively associated neutral faces, *M* (*SD*)	42.11^a^ (13.03)	71.67^a^ (19.61)	60.40^a^ (20.90)
Neutrally associated neutral faces, *M* (*SD*)	44.90^a^ (11.69)	37.58^b^ (18.15)	40.59^b^ (21.42)
*Disapproval (inverted)*			
Negatively associated neutral faces, *M* (*SD*)	55.91^a^ (13.62)	24.03^a^ (18.86)	34.77^a^ (19.94)
Neutrally associated neutral faces, *M* (*SD*)	53.69^a^ (12.61)	56.88^b^ (15.94)	55.81^b^ (21.25)
**Dot-probe task**			
*Experiential rating composite score*			
Negatively associated neutral faces, *M* (*SD*)	42.83^a^ (11.30)	76.98^a^ (17.31)	68.88^a^ (17.51)
Neutrally associated neutral faces, *M* (*SD*)	43.50^a^ (11.58)	29.85^b^ (15.41)	32.36^b^ (15.96)
*Arousal*			
Negatively associated neutral faces, *M* (*SD*)	39.76^a^ (14.73)	71.88^a^ (18.41)	64.68^a^ (18.45)
Neutrally associated neutral faces, *M* (*SD*)	40.95^a^ (13.81)	25.81^b^ (17.03)	30.70^b^ (17.18)
*Valence*			
Negatively associated neutral faces, *M* (*SD*)	41.43^a^ (12.71)	76.76^a^ (19.91)	67.75^a^ (19.74)
Neutrally associated neutral faces, *M* (*SD*)	41.32^a^ (14.20)	27.93^b^ (17.19)	31.43^b^ (17.67)
*Disapproval (inverted)*			
Negatively associated neutral faces, *M* (*SD*)	52.71^a^ (13.92)	17.71^a^ (19.67)	25.79^a^ (18.81)
Neutrally associated neutral faces, *M* (*SD*)	51.77^a^ (15.97)	64.19^b^ (17.44)	65.04^b^ (16.27)

*Means in the same column sharing the same superscript letter do not differ significantly from one another at p ≤ 0.05.*

#### Attention Task

Means and standard deviations of the emotional Stroop index score and RTs of trials with negatively as well as neutrally associated faces of the emotional Stroop task are shown in Table 4. Table 5 presents bivariate Pearson correlation coefficients of different types of maltreatment and the emotional Stroop index score and RTs of trials with negatively as well as neutrally associated faces of the emotional Stroop task. Separate gender- and age-adjusted hierarchical regression analyses were carried out for each of the three indices to examine the unique contributions of different kinds of child maltreatment and relational peer victimization in the prediction of attential biases (Table 6). With respect to the emotional Stroop index score, relational peer victimization made a significant incremental contribution of variance (4%) to the prediction of the score beyond the variance explained by child maltreatment. In the final model [*F*(7, 86) = 2.27, adjusted *R*^2^ = 0.09, *p* = 0.036], however, emotional abuse was the strongest predictor showing a positive association, while peer victimization was negatively associated with the emotional Stroop index score. In the prediction of RTs of trials with negatively associated faces, neither child maltreatment (2%) nor peer victimization (1%) made significant incremental contributions. Here, age was the only significant predictor in the final model [*F*(7, 86) = 14.35, adjusted *R*^2^ = 0.50, *p* < 0.001]. Similarly, age was the strongest predictor for RTs of trials with neutrally associated faces. Though, showing a positive association, peer victimization contributed significantly to the prediction of RTs with neutrally associated faces, explaining additional 3% of the variance, after controlling for the influence of child maltreatment [final model: *F*(7,86) = 11.79, adjusted *R*^2^ = 0.45, *p* < 0.001].

**TABLE 4:** Means and standard deviations on the index scores and reaction times for the emotional Stroop (*N* = 94) and the dot-probe task (*N* = 89).

	*M* (*SD*)
**Emotional Stroop task**	
Emotional Stroop index score	0.44 (79.65)
RTs of trials with negatively associated faces	912.06 (222.24)
RTs of trials with neutrally associated faces	911.61 (217.67)
**Dot-probe task**	
Attentional bias score	–0.34 (17.93)
Orienting index score	–0.26 (18.08)
Disengaging index score	–0.08 (14.43)

**TABLE 5:** Bivariate Pearson correlation coefficients of different types of maltreatment and the indices of the emotional Stroop (*N* = 94) and the dot-probe task (*N* = 89).

Emotional Stroop task	Emotional Stroop index score	RTs of trials with negatively associated faces	RTs of trials with neutrally associated faces
	*r*	*R*	*r*
Emotional abuse	0.25*	0.23*	0.14
Emotional neglect	0.07	0.27**	0.25*
Physical abuse	0.08	0.16	0.14
Sexual abuse	0.13	0.22*	0.18
Peer victimization	-0.16	0.09	0.15

**Dot-probe task**	**Attentional bias score**	**Orienting index score**	**Disengaging index score**
	***r***	***R***	***r***

Emotional abuse	0.08	0.13	–0.07
Emotional neglect	–0.04	–0.06	0.02
Physical abuse	–0.06	–0.05	–0.01
Sexual abuse	–0.15	–0.20	0.05
Peer victimization	0.29**	0.14	0.18

*p < 0.05, **p < 0.01, ***p < 0.001.

**TABLE 6:** Hierarchical multiple regression analysis for the prediction of attentional bias scores in the emotional Stroop task (*N* = 94).

	Variable	β	*R* ^2^	Adjusted *R*^2^	Δ*R*^2^	Δ*F*
**Emotional Stroop index score**						
Step 1			0.05	0.02	0.05	2.14
	Gender	0.10				
	Age	0.11				
Step 2			0.12	0.06	0.07	1.76
	Emotional abuse	0.37*				
	Emotional neglect	–0.09				
	Physical abuse	0.07				
	Sexual abuse	–0.05				
Step 3			0.16	0.09	0.04	4.07*
	Peer victimization	–0.25*				
**RTs of trials with negatively associated faces**						
Step 1			0.51	0.49	0.51	46.47***
	Gender	0.06				
	Age	0.72***				
Step 2			0.53	0.50	0.02	1.06
	Emotional abuse	0.15				
	Emotional neglect	–0.06				
	Physical abuse	0.01				
	Sexual abuse	–0.02				
Step 3			0.54	0.50	0.01	1.96
	Peer victimization	0.13				
**RTs of trials with neutrally associated faces**						
Step 1			0.45	0.44	0.45	37.06**
	Gender	0.02				
	Age	0.69***				
Step 2			0.46	0.42	0.01	0.37
	Emotional abuse	0.01				
	Emotional neglect	–0.03				
	Physical abuse	–0.02				
	Sexual abuse	0.00				
Step 3			0.49	0.45	0.03	5.33*
	Peer victimization	0.22*				

*p < 0.05, **p < 0.01, ***p < 0.001.

*β coefficients of the final models are presented.*

### Dot-Probe

#### Social-Conditioning Paradigm

To test whether the association of the neutral faces with negative and neutral valences was successful, a CS-type × Time of assessment ANOVA using the experiential rating composite score was conducted. The ANOVA revealed significant main effects of CS-type and time of assessment [CS-type: *F*(1,88) = 188.39; *p* < 0.001; η^2^ = 0.682; time of assessment: *F*(2,176) = 52.36; *p* < 0.001; η^2^ = 0.373; ε = 0.69]. Moreover, a significant interaction of CS-type and time of assessment was found [*F*(2,176) = 181.49; *p* < 0.001; η^2^ = 0.673; ε = 0.79]. There were no significant differences in experiential ratings of the neutral faces before the conditioning task [*t*(88) = 0.46, *p* = 0.646]. However, *post hoc t*-tests revealed that experiential ratings of the negatively associated familiar faces were rated significantly more negatively than the neutrally associated familiar faces immediately after the conditioning task as well as after the dot-probe task [post-conditioning: *t*(88) = 16.53, *p* < 0.001; post dot-probe task: *t*(88) = 12.26, *p* < 0.001]. Means and standard deviations of experiential rating scores are presented in Table 3.

#### Attention Task

Means and standard deviations of the index scores are shown in Table 4. Table 5 presents bivariate Pearson correlation coefficients of different types of maltreatment and the indices of the dot-probe task. For each of the three indices separate gender- and age-adjusted hierarchical regression analyses were carried out to investigate the unique proportion of variance accounted for by retrospective reports of child maltreatment and relational peer victimization. As reported in Table 7, relational peer victimization made a significant incremental contribution of variance (8%) to the prediction of the attentional bias score beyond the variance explained by child maltreatment. In the final model [*F*(7,81) = 4.02, adjusted *R*^2^ = 0.19, *p* = 0.001], peer victimization was the strongest predictor with gender and sexual abuse also remaining significant. While sexual abuse showed a negative association, peer victimization and gender were positively associated with the attentional bias score. With respect to the orienting index score, child maltreatment contributed significantly to the prediction (17%) whereas peer victimization did not show a significant incremental contribution of variance (2%). Here, emotional and sexual abuse remained the only significant predictors when entering peer victimization as an additional predictor in the final model [*F*(7,81) = 2.76, adjusted *R*^2^ = 0.12, *p* = 0.013]. Being the strongest predictor, emotional abuse showed a positive association with the orienting index score, while a negative association was found for sexual abuse. For the disengaging index score, neither child maltreatment nor peer victimization showed significant incremental contribution of variance beyond the variance explained by gender [final model: *F*(7,81) = 2.29, adjusted *R*^2^ = 0.09, *p* = 0.035].

**TABLE 7:** Hierarchical multiple regression analysis for the prediction of attentional bias scores in the dot-probe task (*N* = 89).

	Variable	β	*R* ^2^	Adjusted *R*^2^	*ΔR^2^*	Δ*F*
**Attentional bias score**						
Step 1			0.08	0.06	0.08	3.59*
	Gender	0.23*				
	Age	0.06				
Step 2			0.18	0.12	0.10	2.44
	Emotional abuse	0.34				
	Emotional neglect	–0.22				
	Physical abuse	–0.19				
	Sexual abuse	–0.28*				
Step 3			0.26	0.19	0.08	9.03**
	Peer victimization	0.35**				
**Orienting index score**						
Step 1			0.00	0.02	0.00	0.10
	Gender	–0.02				
	Age	0.07				
Step 2			0.17	0.11	0.17	4.11**
	Emotional abuse	0.56**				
	Emotional neglect	–0.29				
	Physical abuse	–0.28				
	Sexual abuse	–0.30*				
Step 3			0.19	0.12	0.02	2.39
	Peer victimization	0.19				
**Disengaging index score**						
Step 1			0.12	0.10	0.12	5.95**
	Gender	0.31**				
	Age	–0.01				
Step 2			0.14	0.08	0.02	0.40
	Emotional abuse	–0.29				
	Emotional neglect	0.10				
	Physical abuse	0.11				
	Sexual abuse	0.03				
Step 3			0.17	0.09	0.03	2.61
	Peer victimization	0.20				

*p < 0.05, **p < 0.01, ***p < 0.001.

*β coefficients of the final models are presented.*

## Discussion

Using a facial emotional Stroop and a facial dot-probe task, we examined the contribution of peer victimization to the prediction of attentional biases beyond experiences of child maltreatment using hierarchical regression analyses in two different samples with varying levels of childhood maltreatment and peer victimization. Consistent with our hypotheses, the present study showed that retrospective reports of relational peer victimization made a significant, incremental contribution to the prediction of attentional biases beyond child maltreatment. In the emotional Stroop task, however, emotional abuse was the strongest predictor for an attentional bias showing a positive association, while peer victimization was negatively associated with the emotional Stroop index score. In the dot-probe task, relational peer victimization was the strongest predictor for an attentional bias. However, when the attentional bias was examined in more detail, peer victimization did not show incremental contributions but again emotional abuse was the strongest predictor for facilitated attention toward negatively associated neutral faces.

In line with prior research, experiences of child maltreatment and peer victimization were related to altered attention and emotion processing in both samples (Field et al., 2001; Pollak and Sinha, 2002; Gibb et al., 2009; Fani et al., 2011; Grant et al., 2011; Dannlowski et al., 2013; van Harmelen et al., 2013; Günther et al., 2015; Iffland et al., 2019; Iffland and Neuner, 2020). Most notably, relational peer victimization predicted attentional biases over and above experiences of physical, sexual and emotional maltreatment within the family context. This is consistent with findings that child maltreatment and peer victimization significantly and independently predict psychopathology (Gren-Landell et al., 2011; Fisher et al., 2012; Sansen et al., 2014). Hence, our findings emphasize on experiences of peer abuse being as detrimental as histories of child maltreatment but also indicate that peer victimization may have a qualitatively different impact on the processing of emotional faces than other adverse childhood experiences. Further, attentional biases as a result of peer victimization may increase the risk of victims of peer abuse for the development of psychopathology (Iffland et al., 2019).

In line with previous results reported by Rosen et al. (2007), retrospective reports of experiences of peer victimization were associated with differentiated color-naming of negatively associated faces compared to neutrally associated neutral faces in the present emotional Stroop task. In prior studies, faster responses in trials with threatening compared to non-threatening cues were reported and have been suggested to indicate more defensively preemptive cognitive processing patterns as attentional avoidance (Newman and McKinney, 2002; Rosen et al., 2007). Accordingly, attentional avoidance in individuals who have experienced peer victimization has also been reported in a previous dot-probe study using emotional and neutral adjectives (Iffland et al., 2019). In the present study, however, RTs of trials with negatively and neutrally associated faces contradicted the suggestion of attentional avoidance in individuals who have experienced peer victimization. While peer victimization is associated with delayed responses to neutrally associated faces, responses to negatively associated faces were not affected by ratings of peer abuse. Hence, the faster responses in trials with threatening compared to non-threatening faces were rather caused by an enhanced interference in trials with neutrally associated faces than by a preemptive and implicit cognitive and emotional reaction to negatively associated faces (Rosen et al., 2007). It may be speculated that the ambiguity of neutrally associated faces is more difficult to process for individuals who have experienced peer victimization. Accordingly, Rudolph et al. (2010) reported increased anticipatory physiological activation in children who had been victimized who were informed that they would be interacting with unfamiliar peers, reflecting a hyper-alertness to social threat. In line with this argument, the present findings in individuals with experiences of peer victimization may also illustrate a hyper-alertness or hyper-vigilance to social threat. That is, individuals who experienced peer victimization may be more likely to anticipate social threat and negative consequences even when confronted with neutral stimuli. Indeed, a previous study using the same set of social-evaluative and neutral videos reported similar psychophysiological responses to both kinds of videos in peer victimized participants (Iffland et al., 2018). Accordingly, peer victimization has been linked to a rather generalized emotion and attention processing style when confronted with different emotions (Iffland et al., 2019). Similarly, childhood bullying has been reported to be associated with paranoid thinking (Campbell and Morrison, 2007; Shakoor et al., 2015) which in turn is linked to a generalized attentional bias toward threatening and neutral stimuli (Jack and Egan, 2016, 2018). Furthermore, attentional avoidance in those who had experienced peer victimization was not supported by the results of the present dot-probe task. Instead, our findings indicated an attentional bias toward negatively associated faces. Accordingly, peer victimization was not associated with avoidance but enhanced interference in an emotional Stroop task in a previous study (Iffland et al., 2019). However, whether this is caused by facilitated attention or by difficulty in disengagement could not be identified in our data (Cisler and Koster, 2010).

It could be that differences in attentional processing styles associated with peer victimization were caused by different demands of the paradigms. Being confronted by a single face indicating social threat as utilized in the emotional Stroop task may elicit different emotion processing than being simultaneously confronted with a face indicating social threat and a neutral face in the dot-probe task. Thus, different attention tasks may address different victim schemas as proposed in the modified social-information-processing model (Rosen et al., 2007). In this model, a victim schema is defined as a cognitive structure comprising an individual’s expectations, cognitions, emotions, and behavior that develop out of repeated patterns of interaction (Rosen et al., 2007). That is, based on previous experiences, an attentional bias toward socially threatening individuals may be adaptive to enable victims of peer victimization to escape from potentially abusive situations when confronted with different individuals as in the dot-probe task (Iffland and Neuner, 2020). However, in situations where no active coping or behavioral resources based on fight or flight stress responses are available, such as being directly confronted with a perpetrator as in the emotional Stroop task, attentional avoidance may reflect an attempt to regulate negative emotions (Williams et al., 1988; Mogg and Bradley, 1998; Bar-Haim et al., 2007; Iffland et al., 2019). Accordingly, attentional avoidance has been reported to be linked to emotional regulation strategies (Mogg et al., 2004; Koster et al., 2005, 2006; Pflugshaupt et al., 2005; Cisler and Koster, 2010). Similarly, Carroll et al. (2019) proposed that attentional biases associated with peer victimization not only enhance threat related attention but also broad selective attention processes toward detecting goal-relevant stimuli. Moreover, it is possible that the use of pictures of faces instead of words in the present tasks have led to different results because of differing underlying mechanisms of processing and their link to semantic memory. Though, comparisons of picture and word versions of the paradigms used in the present study showed similar results so it has been suggested that they can be used equally (Stormark and Torkildsen, 2004; Hester et al., 2006).

Although the present study showed that experiences of peer victimization contribute uniquely and additionally to the prediction of attentional biases, the direction and the magnitude of its influence still remains open. That is, when looking at the specific tasks, the results of the present study were in opposition to results reported in previous studies (Rosen et al., 2007; Iffland et al., 2019). Hence, from an aggregational perspective, it may also be suggested that no association of peer victimization and attentional biases is present. Therefore, conclusions from the results of the present study have to be drawn with caution and further research is needed to specify underlying mechanisms and the impact of potential paradigm peculiarities (e.g., kinds of stimuli, task demands).

With respect to the effects of childhood maltreatment within the family context on attentional processes, as hypothesized, our results were consistent with previous findings indicating that particularly emotional forms of maltreatment are associated with attentional biases in the processing of emotional cues (Pollak et al., 2000; Günther et al., 2015; Iffland and Neuner, 2020). In both the emotional Stroop and the dot-probe task emotional abuse was related to facilitated orientation toward negatively associated neutral faces. Faster recognition of negatively associated faces as well as an attentional bias toward sad facial expressions had previously been associated with more frequent reports of emotional abuse (Günther et al., 2015; Iffland and Neuner, 2020). Similarly, the present results were consistent with previous findings of greater sensitivity in detecting threatening cues from emotionally ambiguous faces in healthy individuals with a frequent proportion of emotional maltreatment (Gibb et al., 2009). Emotional neglect, however, was not linked to attentional bias scores within the present study, although bivariate correlations indicated that experiences of emotional neglect were related to slower color-naming of both negatively and neutrally associated neutral faces in the emotional Stroop task. This finding is in line with associations of emotional neglect with slower detection of negatively and neutrally associated familiar faces in a crowd of unfamiliar faces reported in a previous study using the same stimulus set (Iffland and Neuner, 2020). Hence, it may be that emotional neglect causes generalized interference in reaction to social stimuli, such as emotional and neutral faces. As argued by Iffland and Neuner (2020), limited emotional expressiveness of their parents may result in difficulties in distinguishing between emotional expressions in victims of emotional neglect. This may impede effective recognition and appropriate response to social cues, especially when individuals are confronted with emotionally indistinct facial expressions (Pollak et al., 2000; Iffland and Neuner, 2020). Consistently, physical neglected children showed impaired emotion recognition abilities in prior research (Pollak et al., 2000).

Consistent with Günther et al. (2015), our findings indicated that physical abuse was not associated with attentional biases when controlling for other forms of maltreatment. However, additional research is needed to reconcile the present findings with prior reports of impeded recognition of negative faces, attentional avoidance of threatening faces, and response biases for angry faces in physically abused individuals (Pollak et al., 2000; Pollak and Sinha, 2002; Pine et al., 2005; Iffland and Neuner, 2020). For instance, the stimulus set used in the social conditioning paradigm of the present study may have caused stronger associations of peer victimization and emotional forms with alterations in attention processes. The social evaluative connotation of the stimuli may be stronger related to emotionally abusive experiences than to physical forms of maltreatment. Hence, future studies using other kinds of stimuli may reveal effects of physical abuse on emotion processing, even when controlling for effects of emotional forms of maltreatment.

Contrasting with previous studies examining differentiated effects of maltreatment forms on attention processes (Günther et al., 2015; Iffland and Neuner, 2020), the present findings indicated threat avoidance in individuals reporting more frequent experiences of sexual abuse. Cognitive interference in survivors of childhood sexual abuse with current PTSD has been reported previously (Freeman and Gayle Beck, 2000; Field et al., 2001; Martinson et al., 2013). However, the presence of a diagnosis of PTSD has been shown to have a greater impact on attentional biases than having a history of sexual trauma alone. Further, contrasting with our findings, a recent meta-analytic review promoted a positive relationship between experiences of sexual victimization and attentional bias toward sexual threat stimuli (Latack et al., 2017). While most studies used explicit sexually threatening stimuli, the more subtle, social evaluative stimuli in the present study may have elicited a different processing style. In line with Klein et al. (2019), socially threatening stimuli may be associated with peri-traumatic experiences of victimis of sexuals abuse and therefore suitable to evoke differential processing in these individuals.

Contrasting with previous studies applying facial emotional Stroop or dot-probe tasks, modified versions of these tasks using faces with neutral facial expressions were utilized in the present study. Therefore, comparing our results to previous findings must be done with caution because the present tasks may refer to different cognitive mechanisms. However, our findings suggest that differential processing of threatening information does not rely on actual threatening facial expressions, but mental representations of threat through associations are sufficient to modify attentional processes. At this point, the present study replicated the findings presented by Iffland and Neuner (2020) using different tasks measuring attention in additional samples of healthy individuals. As depicted by the experiential ratings, negatively associated neutral faces were perceived as significantly more negative than the neutrally associated faces increasing the likelihood of an activation of the proposed victim schemas or mental representations of maltreatment (Rosen et al., 2007). Assuming that emotion processing in victims of childhood maltreatment and peer victimization is influenced by mental representations rather than by facial expressions alone (Iffland and Neuner, 2020), encountering potential perpetrators could already trigger a cascade of attentional, emotional, and behavioral processes without the counterpart even having expressed, said, or done anything. Hence, the present study presents an ecologically valid attempt to expand the understanding of information processing in the aftermath of childhood maltreatment and peer victimization. It is possible, though, that the stimulus set may not have been arousing or threatening enough to generate general attentional biases to the negatively associated neutral faces. While clearly and strongly threatening stimuli could mask different processing, it may be assumed that less arousing and more ambiguous stimuli are better suited to evoke differentiated processing in maltreated samples (Pollak and Sinha, 2002; Gibb et al., 2009). In line with this conceptualization, even less arousing and more ambiguous stimuli elicit experience-specific information-processing biases in maltreated children due to adaptively increased sensitivity to signals of danger (Pollak, 2003; Gibb et al., 2009). However, future studies are needed to examine whether the absence of absolute attentional biases in the present study were due to stimulus characteristics or paradigm modifications and to better understand which performances are reflected in the modifications.

To our knowledge, this is the first study that simultaneously investigated different forms of maltreatment in the family context and relational peer victimization as predictors of attentional biases. Extending previous research indicating differential associations between various forms of maltreatment and emotion processing (Pollak et al., 2000; Günther et al., 2015; Iffland and Neuner, 2020), our findings suggest that peer victimization can be considered to be at least as detrimental for emotion processing in adulthood as other forms of childhood maltreatment, which may be a risk factor for the development of psychopathological symptomatology. Moreover, the results emphasize that emotional and social forms of maltreatment have at least the same predictive value concerning attentional biases as sexual and physical abuse. However, further studies using a wide range of paradigms and methods (e.g., eye tracking in addition to reaction times) including additional sets of stimuli (e.g., positive stimuli, physical abuse related stimuli) are needed to examine the specific effects of different forms of maltreatment and peer victimization on information processing.

The present study has several limitations that must be considered when interpreting the results of the present study. Because of the cross-sectional design of the study, conclusions about the causal relationship between child maltreatment, peer victimization, and altered emotion processing cannot be drawn. In addition, the assessment of adverse experiences in family and peer context was based on self-report and retrospective accounts which may be subject to recall biases (Häuser et al., 2011). However, this is a limitation common to the field, as investigating the consequences of the full range of childhood maltreatment often lacks valid alternatives to restrospective reports. Particularly emotional forms of maltreatment and victimization are not reliably documented in child protection service, clinical, or medical records. It has been reported, though, that recall biases in reporting childhood maltreatment were not large enough to invalidate retrospective reports (Hardt and Rutter, 2004). For the purpose of examining causality, future research using longitudinal prospective designs is needed. Furthermore, the generalizability of our findings is limited. The sample was relatively young and highly educated, with participants who were predominantly female. This should be addressed in future studies using larger and more representative samples. Additionally, attentional biases in the present study were affected by individual characteristics. In the emotional Stroop task, age was positively associated with RTs of trials with both negatively and neutrally associated faces supporting previous findings that participants become slower with age (Ashley and Swick, 2009; Agustí et al., 2017; Gajewski et al., 2020). Moreover, in accordance with previous studies (Pfabigan et al., 2014; Campbell and Muncer, 2017; Torrence and Troup, 2018), the dot-probe task revealed differences between genders in processing emotional faces. While women tended to show threat avoidance, men showed an attentional bias toward negatively associated faces, particularly showing a difficulty in disengaging from these faces. Future research is needed to examine influences of individual characteristics as age and gender on attentional processes in association with childhood maltreatment and peer victimization. While mean scores of emotional abuse, physical abuse, and sexual abuse were comparable to mean scores of child maltreatment in a representative sample of the German population (Häuser et al., 2011; Iffland et al., 2013), results may have been influenced by lower levels of emotional neglect. Similarly, variance of the CTQ subscales differed within the present samples. Thus, restricted variability may explain the lack of significant associations for some kinds of maltreatment. Hence, replication of the current findings in samples with more varying levels of childhood maltreatment exposure is desirable. The present study is further limited by its focus on the unique effects of peer victimization and types of child maltreatment on attentional processes. Since different forms of maltreatment are intercorrelated and often co-occur (Häuser et al., 2011), it is likely that interactions between maltreatment types as well as maltreatment types and peer victimization influence emotion processing beyond the unique effects of each. Therefore, future studies should investigate cumulative and interactive effects of types of maltreatment associations. Lastly, it has been documented that childhood maltreatment increases the risk for experiencing adversities later in life (e.g., Dong et al., 2004; Finkelhor et al., 2007). Because exposure to adverse experiences in adolescence and adulthood were not measured in the present study, potential effects of these additional adversities could not be controlled for in our analyses, which should be addressed in future research.

## Conclusion

Prevalence rates of peer victimization in school children of 10–20% are alarming (Rudolph et al., 2010), particularly with respect to the strong associations of peer victimization and psychopathology (e.g., Storch et al., 2005). The current study contributes to a better understanding of potential paths linking peer victimization and psychopathology by expanding previous reports of altered processing of incoming emotional information in the aftermath of childhood maltreatment within the family context (e.g., Wells et al., 2014; Günther et al., 2015; Iffland and Neuner, 2020). It has been suggested that cognitive alterations in victims of maltreatment contribute to inadequate and maladaptive responding to social interactions, setting individuals at risk for further victimization and later psychopathology (Rosen et al., 2007; Masten et al., 2008; Fani et al., 2011; Wells et al., 2014). In addition to supporting prior studies that indicate that particularly emotional maltreatment is associated with alterations in attentional processes (Iffland and Neuner, 2020), the results of the present study indicate that peer victimization leaves additional cognitive scars that may contribute to a broad range of psychopathology. A better understanding of the specific characteristics in the processing of emotional stimuli in the wake of peer victimization and other forms of childhood maltreatment is therefore needed to address short and long term consequences and treatment offers for victims.

## Data Availability Statement

The datasets generated for this study are available on request to the corresponding author.

## Ethics Statement

The studies involving human participants were reviewed and approved by Ethics Committee of Bielefeld University. The patients/participants provided their written informed consent to participate in this study.

## Author Contributions

BI participated in the conception and design of the study, collected the data, performed the statistical analyses and interpretation of findings, and drafted the manuscript. FN participated in the conception and design of the study, made substantial contributions to the statistical analyses and interpretation of findings, and helped to draft and revise the manuscript. Both authors read and approved the final manuscript.

## Conflict of Interest

The authors declare that the research was conducted in the absence of any commercial or financial relationships that could be construed as a potential conflict of interest.

## Publisher’s Note

All claims expressed in this article are solely those of the authors and do not necessarily represent those of their affiliated organizations, or those of the publisher, the editors and the reviewers. Any product that may be evaluated in this article, or claim that may be made by its manufacturer, is not guaranteed or endorsed by the publisher.
